# Sustained training with novel distractors attenuates the behavioral interference of emotional pictures but does not affect the electrocortical markers of emotional processing

**DOI:** 10.3389/fpsyg.2024.1322792

**Published:** 2024-02-07

**Authors:** Vera Ferrari, Francesca Canturi, Andrea De Cesarei, Maurizio Codispoti

**Affiliations:** ^1^Department of Medicine and Surgery, University of Parma, Parma, Italy; ^2^Department of Psychology, University of Bologna, Bologna, Italy

**Keywords:** emotional scenes, training, behavioral interference, LPP, Alpha-ERD

## Abstract

**Introduction:**

Research has recently shown that behavioral interference prompted by emotional distractors is subject to habituation when the same exemplars are repeated, but promptly recovers in response to novel stimuli. The present study investigated whether prolonged experience with distractors that were all novel was effective in shaping the attentional filter, favoring stable and generalizable inhibition effects.

**Methods:**

To test this, the impact of emotional distractors was measured before and after a sustained training phase with only novel distractor pictures, and that for a group of participants depicted only a variety of neutral contents, whereas a different group was exposed only to emotional contents.

**Results:**

Results showed that emotional interference on reaction times was attenuated after the training phase (compared to the pre-test), but emotional distractors continued to interfere more than neutral ones in the post-test. The two groups did not differ in terms of training effect, suggesting that the distractor suppression mechanism developed during training was not sensitive to the affective category of natural scenes with which one had had experience. The affective modulation of neither the LPP or Alpha-ERD showed any effect of training.

**Discussion:**

Altogether, these findings suggest that sustained experience with novel distractors may attenuate attention allocation toward task irrelevant emotional stimuli, but the evaluative processes and the engagement of motivational systems are always needed to support the monitoring of the environment for significant cues.

## 1 Introduction

In our daily life, whether we are exploring the environment in search of something or someone, or navigating the web, it often happens that we run into important stimuli that involuntarily capture attention and interrupt ongoing goal-directed activities. Selective attention enables individuals to commit cognitive resources to significant elements in the visual environment while filtering irrelevant sensory input (Egeth and Yantis, 1997; Folk, 2015). It is well established that there are certain classes of stimuli that, when they are distractors, are more resistant to inhibition than others (Folk, 2015). Emotional stimuli are an example, especially those depicting sexual or blood/wound contents (Most et al., 2007; De Cesarei and Codispoti, 2008), which have a high attentional priority. Research has shown that emotional pictures engage attentional resources, and hence disrupt performance (i.e., elongate reaction time) in a variety of concurrent tasks (Hartikainen et al., 2000; Schimmack, 2005; Most et al., 2007; De Cesarei and Codispoti, 2008; Ferrari et al., 2017).

According to several studies, the viewing of emotional pictures activate corticolimbic motivational systems (appetitive and aversive) that, in turn, enhance attention allocation to efficiently process the stimulus itself (Öhman, 1992; Hamm et al., 2003; Bradley, 2009; Gottlieb, 2012; Codispoti et al., 2016; Mulckhuyse, 2018). In terms of electrocortical activity, it is well established that emotional pictures (pleasant and unpleasant) elicit a larger late positive potential (LPP) and Alpha event-related desynchronization (Alpha ERD) than neutral images (Schupp et al., 2006; De Cesarei and Codispoti, 2011; Ferrari et al., 2020; Schubring and Schupp, 2021; Ferrari et al., 2022), and these cortical modulatory effects have been interpreted as reflecting both the engagement of attentional resources by emotional stimuli and the activation of motivational systems (Schupp et al., 2006; Lang et al., 2008; Ferrari et al., 2011; Weinberg and Hajcak, 2011; for a review focusing on similarities and differences between these measures see Codispoti et al., 2023).

It has been suggested that being repeatedly exposed to irrelevant events leads to more efficient filtering of those events (Kelley and Yantis, 2009; Vecera et al., 2014). Mere stimulus repetition is also effective in attenuating attentional capture by emotional distractors (Codispoti et al., 2016; Ferrari et al., 2022). However, emotional interference recovered when novel stimuli were presented after the habituation phase, indicating that the filtering mechanism was finely tuned regarding the specific stimulus used throughout habituation and did not apply to similar emotional exemplars, such as those presented in the novel phase (Wendt et al., 2011; Codispoti et al., 2016).

Research seems to indicate that attentional capture by emotional distractors can be attenuated even with novel stimuli (i.e., never repeated) by increasing the overall distractor frequency (Micucci et al., 2020). When participants were rarely exposed to distractors (20% of total trials, 10% emotional, 10% neutral), emotional pictures captured more attentional resources compared with neutral images, causing a behavioral interference (RT slowdown) with the ongoing task. However, this RT emotional interference (emotional vs. neutral) decreased when the overall frequency of distractors increased (80% of total trials, 40% emotional, 40% neutral). The emotional interference was attenuated by distractor frequency even when rare emotional distractors appeared among frequent neutral distractors (80% of total trials, 10% emotional, 70% neutral). In the same study (Micucci et al., 2020), distractor frequency (using entirely novel stimuli), moreover, did not attenuate the affective modulation of the late positive potential, suggesting that the high occurrence of distractors does not proactively prevent the processing of emotional task-irrelevant cues. Distractor repetition (same exemplar) was, on the other hand, effective in reducing both behavioral interference as well as the LPP modulation (Ferrari et al., 2022) when distractors were task-irrelevant pictures presented in peripheral vision while a central discrimination task was being performed.

These series of studies support the hypothesis that attentional capture prompted by emotional stimuli is a flexible mechanism that can be temporarily affected by contextual factors (distractor frequency) and learning (habituation), with effects at different response levels (i.e., behavioral interference and LPP). Here, we aim to extend the assessment of experience-related factors that can be effective in attenuating the interference of emotional distractors, with a specific question regarding whether the experience with distractors that accumulates over time is effective in shaping the attentional filter, favoring stable and generalizable inhibition effects. In other words, can attentional capture by novel and emotional distractors be attenuated through prolonged experience over time? We already know that experience with the same distractors (same exemplars repeated multiple times) is only effective in attenuating the interference of those distractors, but it does not generalize toward new, subsequently presented, distractors. The literature, on the other hand, indicates that when the trained distractors are variable, the attentional filter encodes multiple features and encompasses broad representations, with the advantage of generalizing suppression more easily beyond the specific trained exemplars to other potential novel stimuli (Dixon et al., 2009; Kelley and Yantis, 2009, 2010; Vatterott and Vecera, 2012; Vatterott et al., 2018). Therefore, the present study introduced sustained experience with distractors that were all new (novel exemplars) in that they were never repeated throughout the study, and tested whether this experience affected the emotional interference of subsequently appearing distractors. Thus, similar to previous studies, emotional and neutral pictures consisted of task-irrelevant stimuli presented in peripheral vision while a central discrimination task was being performed. After an initial block of trials (pre-test) in which the enhanced interference of emotional distractors was measured in terms of behavioral and brain responses, the same task continued to be performed in a practice phase, which had the goal of providing a sustained and prolonged experience with distractors (twice as many trials as the pre-test). A final block (post-test) was then introduced to assess the amount of attentional capture by emotional pictures compared to the pre-test. Thus, the main question of the study was whether emotional interference may benefit from sustained experience with novel distractors. The second question concerns the specific content of distractors during the practice phase. Do observers need specific practice (experience) with emotional distractors in order to effectively inhibit their processing? Given the obligatory nature of emotional processing, we may expect that general experience with distractors is not sufficient to efficiently shield the attentional set from distraction. More sustained experience with wholly novel emotional distractors might help tune a specific filter based on the perceptual similarities within the semantic category (e.g., erotica), creating a situation that is more similar to that of repeated distractors (habituation paradigm, Ferrari et al., 2022) which is highly effective in preventing emotional interference. The goal of the present study was to investigate whether the filtering becomes effective with general experience with simply neutral (non-emotional) distractors, or whether specific emotional practice is needed to prompt a stable distractor inhibition mechanism. Thus, one group of subjects underwent a practice phase with solely neutral distractors, whereas a different group had experience with distractors that were all emotional (a between-subject design, where only the type of distractors used in the practice phase differed).

Distractor filtering may occur at various stages of processing. Therefore, besides behavioral interference, indexed by RTs, we also examined two cortical indexes of emotional processing, the LPP and Alpha-ERD, to better clarify at which stage the filter can operate. Sustained exposure to task-irrelevant distractors may affect the activation of motivational systems, preventing the cascade of perceptual and motor responses that are typically prompted by the detection of emotional stimuli. Alternatively, motivational systems might continue to be engaged after an extensive exposure to novel distractors to support some fundamental sensory processing, without necessarily interfering with the ongoing task performance. Although a reduction in emotional interference can be predicted by both these scenarios, emotional effects at the cortical level may reveal the extent to which frequent distractors are actually ignored.

Research has shown that Alpha-ERD emotional modulation is unaffected by top-down factors, such as task-related processes (Schubring and Schupp, 2019; Codispoti et al., 2023), or picture repetition (Ferrari et al., 2015; 2020; 2022; Schubring and Schupp, 2021). The affective modulation of the LPP is affected by stimulus repetition (Codispoti et al., 2007; Mastria et al., 2017), and there is evidence of stronger habituation when pictures are outside the attentional focus, and behave as distractors (Ferrari et al., 2022). Similarly, a prolonged experience with novel distractors may result in a narrowing of the attentional focus, with an effect also at the level of the LPP and Alpha ERD modulation.

While the topography of the overall LPP (regardless of picture content) does not vary depending on the hemifield of picture presentation, alpha activity has been repeatedly shown to decrease more in the hemisphere contralateral to the stimulus/target location (Bacigalupo Izquierdo and Luck, 2019; Murphy et al., 2020; Arana et al., 2022; Ferrari et al., 2022). Therefore, in the present study, we examined whether sustained experience with novel distractors resulted in reduced contralateral Alpha-ERD, possibly reflecting distractor filtering associated with an enhanced attentional focus on (central) target processing.

In previous studies, these cortical and behavioral (RTs: emotional vs. neutral interference) measures were used interchangeably (to some extent) to examine the effects of emotion on attention (MacNamara and Hajcak, 2009; Weinberg et al., 2021), and it has also been suggested that LPP and emotional interference are associated with each other during affective picture processing (Weinberg and Hajcak, 2011; Weinberg et al., 2021). A further aim of the present research is to explore the relationships among these neural and behavioral markers of emotional processing.

## 2 Method

### 2.1 Participants

A total of fifty-four (27 females; mean age = 25.2 years, SD = 4.6) healthy students participated as volunteers in the study, and all signed the informed consent before starting the experiment. Twenty-six students were randomly assigned to the “neutral training” protocol, and twenty-eight to the “emotional training” protocol. Because of technical problems, data from one participant in the neutral practice protocol were not included in the overall analysis. The study was approved by the Ethical Committee of the University of Parma. All participants had normal or corrected-to-normal visual acuity. We estimated sample size using GPower* (Faul et al., 2007), aiming to determine the number of participants necessary, in an ANOVA with one between-participant factors with two levels (neutral vs. emotional training group) and two within-participant factors (each one with two levels, Phase and emotional vs. neutral), to observe an effect size of at least η^2^_p_ = 0.07 (medium effect size, Cohen, 1988), with 0.05 alpha-error probability, 80% power, and a correlation among repeated measures of 0.6. This analysis yielded 24 as the result.

### 2.2 Material

A total of 900 pictures of natural scenes were selected from the International Affective Picture System (IAPS; Lang et al., 2008), and from public domain pictures available on the Internet. Of these pictures, 30 depicted pleasant contents (erotica), 30 unpleasant contents (injured bodies) and 60 showed people in neutral contexts, and all of these were always presented in the pre-test or in the post-test (all pictures were counterbalanced across conditions). 480 stimuli depicted neutral contents from a wide variety of semantic categories, such as objects, animals, means of transportation, and outdoor or indoor scenes. Of these, 180 pictures were used as neutral fillers in the pre- and post-test for both groups (neutral and emotional groups). The remaining 300 were used for the neutral training group. For the emotional training group only, 300 pictures depicting emotional contents (erotica and injured bodies, equally distributed) were selected from public domain pictures available on the Internet to be presented during the practice phase.

Pictures of natural scenes served as distractor stimuli and were positioned to either the left or right of a central Gabor patch (sinusoidal gratings with a Gaussian envelope). The distance between the inner edge of the distractor image and the center of the Gabor patch was 4°.

The Gabor patch subtended a 5.3° × 5.3° visual angle and it could be horizontally or vertically oriented. Gabor patches were generated using custom MATLAB software by overlapping two distinct Gabor patches with the same frequencies but different orientation (0.94 and 9.4 cycles per degree of visual angle, respectively). Stimuli were displayed on a gray background.

Stimuli were presented on a 16-in monitor at 1024 × 768 resolution and at a refresh rate of 120 Hz. Stimulus presentation and data collection were performed using E-Prime software (Schneider et al., 2002).

### 2.3 Procedure

Upon arrival at the laboratory, participants signed an informed consent form. They were then seated in a recliner in a small, sound-attenuated, dimly lit room, and the EEG sensor net was attached. Participants sat in front of the computer monitor with their head supported by a chinrest. For all subjects the distance between their eyes and the monitor was 51 cm.

The experimental session consisted of three main phases: an initial Pre-Test, a Training Phase, and a final Post-Test. The Pre-Test and Post-Test phases were identical and consisted of 300 trials each in which distractors appeared randomly in 50% of the trials (*n* = 150). Of these distractors, 30 pictures depicted emotional (half pleasant and half unpleasant) contents, 30 neutral people, and the remaining 90 distractors were pulled from the neutral filler category. The training phase consisted of 600 trials, in which, again, distractors appeared in 50% of the trials and were exclusively neutral scenes for the neutral training group, and only emotional distractors for the emotional training group. Across participants of both groups, four stimulus sets with different pictures were equally rotated across conditions (pre-test and post-test) to make the results more generalizable in terms of stimulus exemplars.

**Figure 1:**
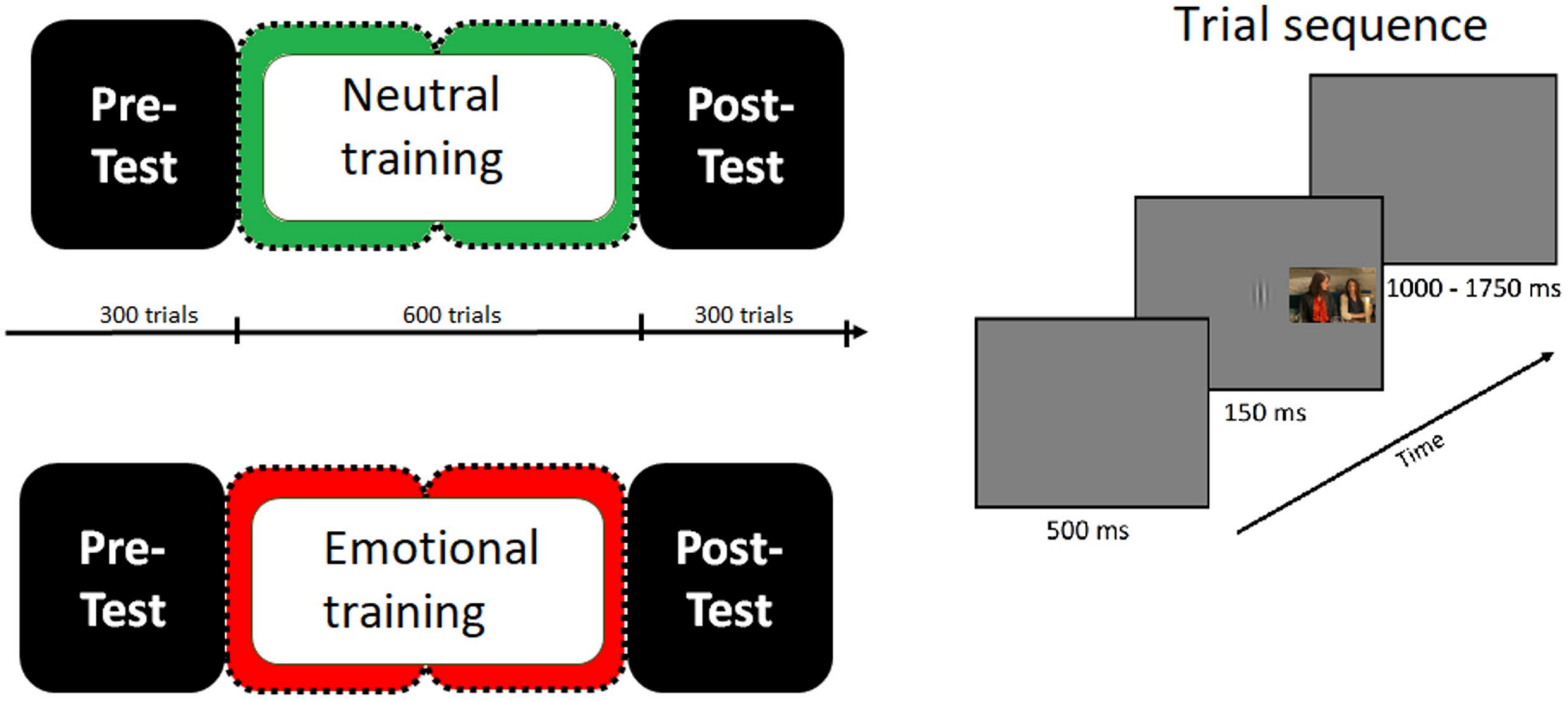
Schematic diagram showing the sequence of events in the present study. The trial sequence was the same over the whole experiment, with an initial dark-gray blank screen appearing for 500 msec, followed by a Gabor patch (target stimulus) presented for 150 msec. In half of the trials with a random occurrence, one picture of a natural scene appeared as a distractor stimulus simultaneously with the Gabor patch, flanking it to the left or right, and stayed on the screen until the Gabor patch disappeared. Participants were instructed to focus their attention on the Gabor patch and to determine its orientation (vertical or horizontal) by pressing one of two buttons while ignoring the distracting scenes. In the pre-test as well as in the post-test distractors were pictures depicting both emotional and neutral contents. In the practice phase, distractors were only neutral pictures for group 1 or only emotional pictures for group 2.

Figure 1 shows the trial and the sequence of events of the experimental paradigm. In each trial, a Gabor patch appeared in the center of the screen for 150 msec. The participant’s task was to determine, as quickly and accurately as possible, whether the Gabor patch was vertical or horizontal by pressing the corresponding keys with the index finger of the dominant hand. The intertrial interval was variable (1,000, 1,550, or 1,750 msec) and consisted of a gray screen. During this period, behavioral responses to the orientation task were collected. In distractor-present trials, a distractor picture was presented simultaneously with the Gabor patch, appearing equally often in the left or right visual field. Participants were explicitly informed that there would be a distractor in some trials and that it should be ignored. The task remained the same throughout the whole experiment. Before the beginning of the experiment, participants performed a short practice session (150 trials), in order to familiarize themselves with the task. Between each block a 5-min break was given. The experiment lasted for approximately 52 min.

### 2.4 EEG recording and processing

**Figure 2:**
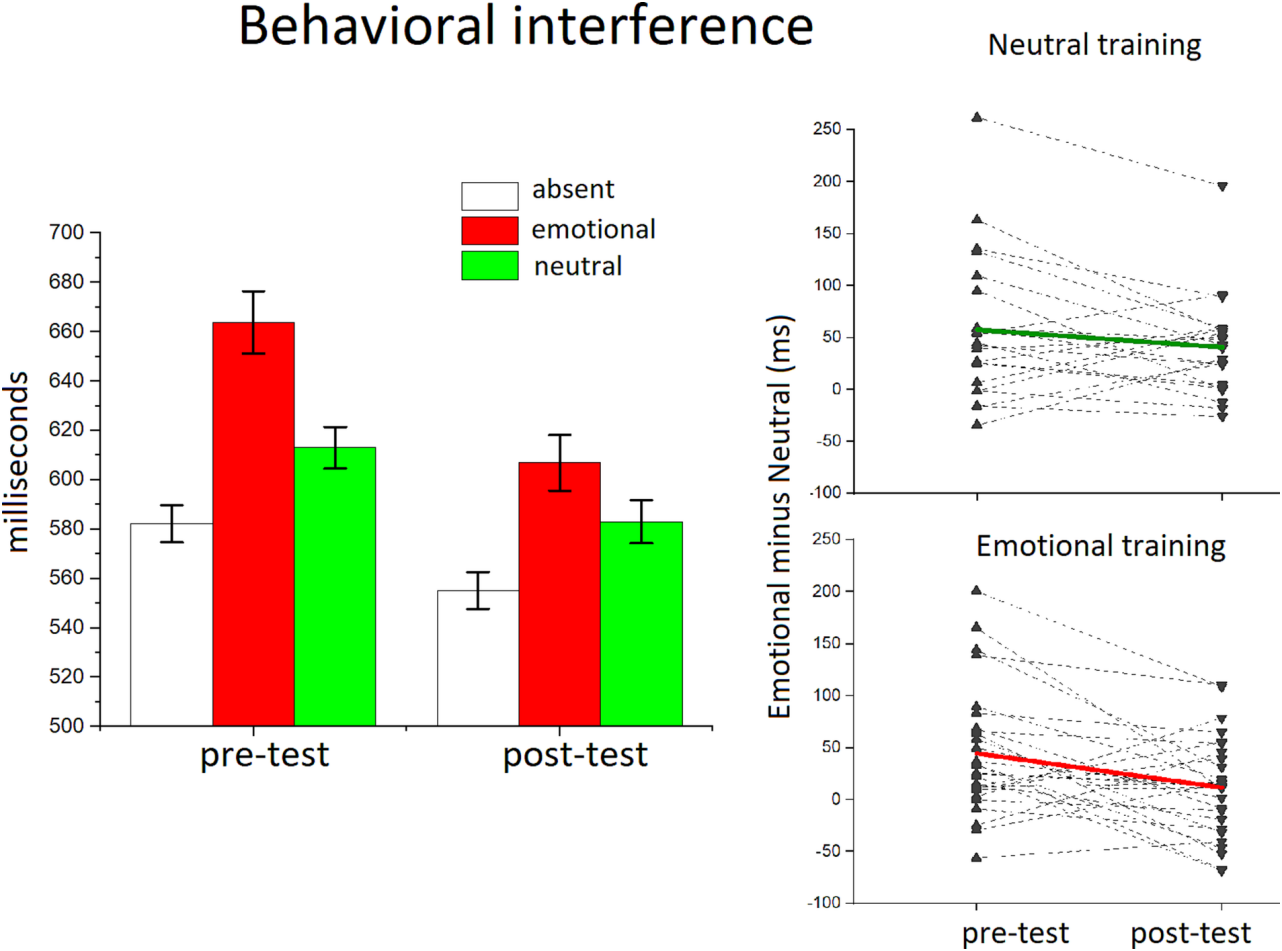
The effects of distractor occurrence on the LPP amplitude. **(A)** Grand-averaged ERP waveforms (average across the sensor cluster) for emotional and neutral distractors in the pre-test block. Insets show the scalp topography (450–900 msec) of the difference in the LPP between emotional and neutral distractors. **(B)** The bar graphs show the mean of the LPP amplitude (window 450–900 ms) for distractor-absent, emotional, and neutral distractors in the pre- and the post-test.

Electroencephalogram (EEG) was recorded at a sampling rate of 1,000 Hz using a 59 channel Electro-Cap connected to a SA Instrument CO (San Diego, CA) UF-64/72BA amplifier and in-house developed software. Impedance of each sensor was kept below 10 kΩ. Eye movements were recorded at a sampling rate of 500 Hz from two bipolar couples of electrodes, one pair placed 1 cm above and below the right eye, and the other 1 cm from the external corner of both eyes. Both EEG and ocular signal were on-line filtered from 0.01 to 100 Hz. E-prime software synchronized the presentation of the stimuli and triggered EEG recording in each trial. Off-line analysis was performed using Emegs (Peyk et al., 2011). First, eye movements were subtracted from the EEG on a trial-by-trial basis, based on the data from the monopolar horizontal and vertical EOG, using a regressive procedure (Gratton et al., 1983). Data were low-pass filtered at 30 Hz. Trials and sensors containing artifacts were detected through a statistical procedure (Junghofer et al., 2000). In each trial, if a high number of neighboring bad sensors was present, then the whole trial was discarded; for the remaining trials, sensors containing artifacts were replaced by interpolating the nearest good sensors. The percentage of good trials was 85%, and this percentage did not significantly change across blocks or conditions. Finally, data were re-referenced to the average of all channels. The average of the 200 ms pre-stimulus baseline was subtracted to the obtained waveform. Processed data were averaged for each Phase (Pre-test and Post-test) and Trial type (distractor absent; emotional and neutral distractors). ROI and time interval of interest were identified both through visual inspection and according to previous studies (Micucci et al., 2020; Ferrari et al., 2022). The LPP was scored as the average of the ERP waveform in the 450 and 900 msec period after stimulus onset at the centro-parietal sensor group (see Figure 2 for the scalp topography of the LPP emotional modulation).

**Figure 3:**
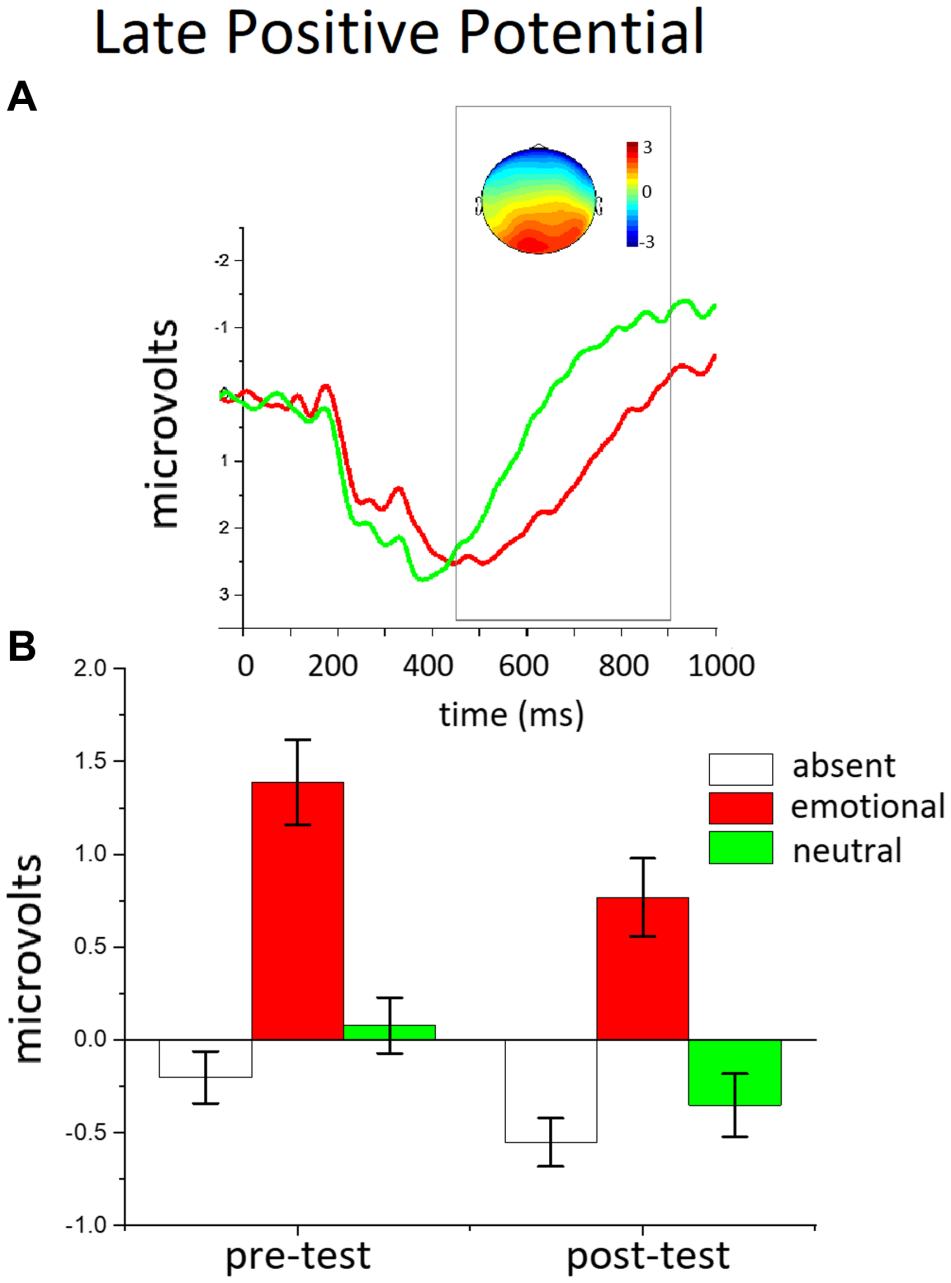
The effects of distractor occurrence on the Alpha-ERD. **(A)** Time-frequency plot of the emotional-neutral difference at occipitotemporal sensor sites, regardless of distractor laterality. **(B)** Topography of alpha power changes (450–900 ms window) for emotional and neutral distractors appearing on the left or on the right of the visual hemifield. **(C)** The bar graphs show the mean of the alpha power changes (window 450–900 ms) for distractor-absent, emotional and neutral distractors in the pre- and the post-test.

For time-frequency analysis, no low-pass filtering was applied on the EEG signal, but the correction of eye movements, as well as the artifact detection and sensor interpolation, was similar to the ERP analysis. Data were convolved using complex Morlet’s wavelet, varying in time and frequency with a Gaussian shape. The time frequency analysis was performed on single trial data using FieldTrip software through EMEGS (Peyk et al., 2011). The Morlet wavelet had a Gaussian shape, where the f/SD(f) ratio was set to 7, and the number of wavelet cycles was set to 5 (Tallon-Baudry et al., 1997). The range of analysis was from 4 to 80 Hz and analysis was performed in time windows from 1,000-ms before picture onset to 1,500 ms after picture onset in steps of 10 ms. As frequency resolution is maximal for low frequencies and minimal for high frequencies (Roach and Mathalon, 2008), the step between successive frequencies varied linearly from 0.5 Hz for the lowest frequencies to 5 Hz for the highest frequencies. All data were baseline corrected by subtracting the average alpha power of the pre-stimulus baseline (− 300 to −100 ms) from each data point. The baseline was calculated slightly earlier than stimulus onset to avoid the burst of oscillatory activity that starts before the onset of the stimulation, due to the artifact of the filter algorithm (Herrmann et al., 2005). The resulting event-related change in total power values (relative to baseline) are in decibels (dB) (Delorme and Makeig, 2004). Analyses of Alpha-ERD (8–14 Hz) were performed on a bilateral occipito-temporal sensor group (see Figure 3) over the same temporal window of the LPP (450–900 ms).

### 2.5 Data analysis

RT and EEG analyses were performed only on accurate trials (overall accuracy 96,4%), that is, when the orientation of the central gabor was correctly detected. For each participant, phase, and trial type, RTs above or below 3 SDs from the mean were discarded as outliers. Considering trials exclusion due to EEG artifacts or behavioral errors/outliers, the EEG signal (both LPP and Alpha-ERD) was analyzed on average over 24 trials per condition.

The same statistical design was applied to all measures analyzed in this study, with two within-subject factors: Phase (2: Pre-test, Post-test) and Trial type (3: distractor absent, emotional, neutral); one between-subject factor represented by the two groups of participants who underwent a different training protocol, one with entirely neutral distractors, the other with all distractors containing emotional content (training type). For each ANOVA test, we reported the partial η^2^ squared statistic (η^2^_p_) indicating the proportion of variance that is explained by experimental conditions over the total variance.

## 3 Results

### 3.1 Behavioral data

Figure 2 illustrates the behavioral interference as a function of distractor content. The ANOVA performed on the whole statistical design (Phase x trial Type x Group) revealed a main effect of trial type *F*(2,50) = 62.545, *p* < 0.0001, η^2^_p_ = 0.714, showing an expected RT slowdown with the occurrence of all distractor pictures, compared to distractor absent trials *F*s(1,51) > 76; ps < 0.0001, η^2^_p_ > 0.741. Distractor interference was enhanced for emotional compared to neutral distractors, *F*(1,51) = 30.605, *p* < 0.0001, η^2^_p_ = 0.375.

There was also an overall decrease of RT across the two phases *F*(1,51) = 14.854, *p* < 0.001, η^2^_p_ = 0.226, and the interaction phase x trial type *F*(2,50) = 8.482, *p* < 0.001, η^2^_p_ = 0.253 indicated that this decrement that followed the training phase significantly affected the RT emotional interference, which was smaller in the post-test compared to the pre-test (phase x emotional vs. neutral distractors, *F*(1,51) = 13.161; *p* < 0.001, η^2^_p_ = 0.205). Although reduced, emotional interference was still highly significant in the post-test, *F*(1,51) = 13.198, *p* < 0.001, η^2^_p_ = 0.206. The between-subject factor that refers to the different distractor category (all neutral or all emotional) used during the training phase did not show any significant effect, suggesting that the attenuation of emotional interference after the sustained training session was not specifically related to the type of experience with distractor content [2 × 3 × 2, *F*(3,50) = 2.359, *p* > 0.05, η^2^_p_ = 0.09]. Moreover, a specific test on the affective modulation, that is, on the difference between emotional and neutral distractors, with the two factors, phase x group (training type), revealed no significant interaction effect, *F*(1,51) < 0.1, *p* = 0.454, η^2^_p_ = 0.011.

The ANOVA on accuracy did not reveal any significant effects involving the three factors, or their interactions.

### 3.2 Late positive potential (LPP)

Figure 3 illustrates the LPP enhancement for emotional, compared to neutral, distractors, over the centro-parietal region. The ANOVA in the LPP window revealed a main effect of Trial Type, *F*(2,50) = 37.928, *p* < 0.0001, η^2^_p_ = 0.603, and of Phase, *F*(1,51) = 13.742, *p* < 0.001, η^2^_p_ = 0.212. Distractor occurrence [any distractor, *F*s(1,51) > 7.591, ps < 0.01, η^2^_p_ > 0.130] prompted a significant increase in the LPP magnitude, compared to distractor absent trials, and the largest positivity was found for emotional distractors, compared to neutral people, *F*(1,51) = 54, *p* < 0.0001, η^2^_p_ = 0.515. Although over time (pre-test vs. post-test) the overall LPP amplitude was attenuated, this decrease did not differ as a function of distractor content [phase x trial type, *F*(2,50) <1, *p* = 0.283, η^2^_p_ = 0.49], indicating that the LPP affective modulation (difference between emotional and neutral distractors) was unaffected by the extended practice with novel distractors. Accordingly, the type of distractors used in the training did not prompt any difference in the LPP modulation between the pre- and the post-test either [phase x trial type x group, *F*(2,50) = <1, *p* = 0.883, η^2^_p_ = 0.005]; in fact, the two groups of participants showed no significant difference in cortical modulation between the two phases, regardless of what kind of practice they had carried out with distractors.

### 3.3 Brain oscillations: Alpha-ERD

**Figure 4:**
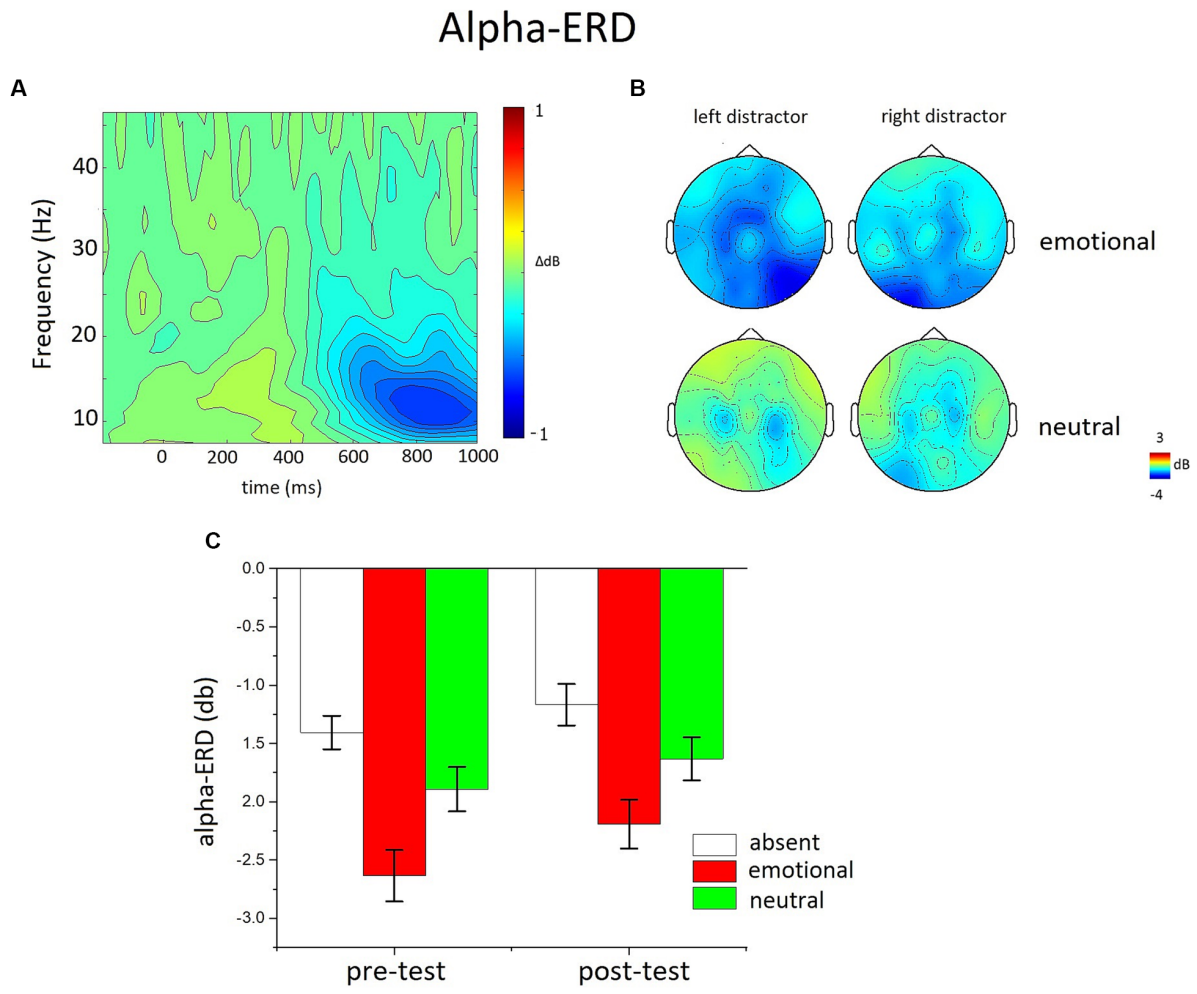
RTs in the discrimination orientation task for each trial type. Behavioral interference is plotted as a function of block, showing that the RT slowdown was maximum with the occurrence of emotional distractors, compared with neutral distractors or trials with no distractors. The emotional interference was clearly attenuated in the post-test. Error bars show ±1 SEM calculated within participants using the method of O’Brien et al. (2014). The inset shows the single subject plots of the emotional interference (emotional minus neutral) in the pre and post-test for each training group.

Figure 4 illustrates Alpha ERD as a function of distractor content and position (left and right hemifield). The power in the Alpha band was clearly reduced compared to the baseline when a peripheral distractor was present compared to absent, and this desynchronization was enhanced for emotional, compared to neutral, pictures in the pre-test, as well as in the post test.

The ANOVA in the alpha band revealed a significant modulation as a function of trial type, *F*(2,50) = 19.968, *p* < 0.0001, η^2^_p_ = 0.444, with the largest desynchronization for trials with the occurrence of emotional distractors, and the smallest for distractor absent trials. Alpha-ERD for neutral distractor trials was significantly larger compared to distractor absent trials, *F*(1,52) = 17.297, *p* < 0.001, η^2^_p_ = 0.253, and significantly smaller compared to emotional trials, *F*(1,52) = 17.727, *p* < 0.001, η^2^_p_ = 0.254. The overall Alpha-ERD was slightly attenuated in the post-test compared to the pre-test, *F*(1,51) = 5.144, *p* < 0.05, η^2^_p_ = 0.092, but the magnitude of this effect did not differ across trial type conditions. The between-subject factor (training type) was not involved in any significant interaction.

An additional analysis focused on the contra/ipsi lateral Alpha-ERD to distractor-present vs. distractor-absent trials, in order to evaluate whether the alpha activity in response to lateralized distractors was affected by the training. The distractor contralateral alpha activity (distractor minus absent) did not vary between the pre-test and the post-test, *F*(1,48) = 1.86, *p* = 0.179, η^2^_p_ = 0.037. Similarly, the distractor ipsilateral alpha activity (distractor minus absent) did not vary between the pre-test and the post-test, *F*(1,48) = 2.28, *p* = 0.137, η^2^_p_ = 0.045.

### 3.4 Cortical (LPP and Alpha-ERD) and behavioral interference: between-subject correlations

In order to directly assess the relationship between different indices of emotional processing (behavioral interference, LPP, and Alpha-ERD), Pearson’s correlation coefficients were calculated using the difference scores (emotional minus neutral), separately for the pre- and the post-test. None of the correlations were significant in the pre-test (−0.18 > rs < 0.07, ps > 0.19) or in the post-test (−0.25 > rs < 0.19, ps > 0.06).

## 4 Discussion

The goal of the present study was to investigate the effects of training on the emotional interference of novel distractors depicting natural scenes. The extended experience with distractors throughout several blocks of trials prompted an attenuation of emotional interference between the post-test and the pre-test. This effect was independent of the type of experience with distractor content, since it occurred to the same extent in both groups of participants, for those whose training involved neutral distractors as well as for those who were exposed to emotional pictures during training. Unlike the behavioral interference, the affective modulation of both cortical indexes of emotional processing, the late positive potential and the Alpha-ERD, was unaffected by the amount and type of distractor exposure.

Previous repetition studies reported emotional distractor suppression after several repetitions of the same exemplars (Ferrari et al., 2022); here, the emotional interference was reduced over trials despite distractors consisting of a variety of novel natural scenes that were never repeated across the experiment. These results are in line with what was found in a similar paradigm in which the exposure to novel distractors varied as a function of distractor frequency occurrence (Micucci et al., 2020), suggesting that sustained experience with distractors can affect emotional capture even when distractors are novel stimuli (never repeated). A possible factor accounting for these effects could be the narrowing of spatial attention: Is the decline of emotional interference due to a specific inhibition of any sensory stimulus appearing in the distractor locations, preventing the identification of the affective category of the stimulus and the consequent engagement of motivational systems? In this respect, findings from cortical measures help to rule out this hypothesis. Indeed, the emotional nature of the distractors modulated both LPP and Alpha-ERD without showing any training effect, indicating that the reduction in the emotional interference effect cannot be explained in terms of a spatially specific inhibition of the distractor locations. Consistently, we did not find a reduction in the controlateral Alpha-ERD between the pre- and the post-test. These findings suggest that repetitive experience attenuates emotional interference, but it does not proactively prevent the processing of emotional distractors. A similar pattern of results was observed when the exposure to distractors was manipulated through distractor frequency occurrence (Micucci et al., 2020). Moreover, these findings are not consistent with a mere attention-capture interpretation of the affective modulation of the LPP and of Alpha-ERD, but provide further support that these cortical markers reflect the engagement of corticolimbic motivational systems in a mandatory fashion, even when further allocation of attention to emotional stimuli is attenuated.

It should be noted that while in previous studies emotional interference disappeared after a few distractor repetitions (Codispoti et al., 2016; Ferrari et al., 2022), here extensive experience with distractors determined a decrease in this effect which, however, continued to persist in the post-test, after over 900 trials. Moreover, it is well established that after substantial affective habituation, the occurrence of new stimuli in a final block (novel phase) was sufficient to reinstate an emotional effect (dishabituation) on several orienting measures (i.e., heart rate, skin conductance, pupil dilation, LPP; see also emotional interference). These findings, together with the somewhat weak effects of training with novel distractors observed in the present study, indicate that it is likely that the orienting mechanism is tuned on the specific exemplar that we directly experience, without generalization that spreads to the entire semantic category to which natural scenes belong, or to simple features that are shared between scenes, e.g., blood in unpleasant pictures (Gati and Ben-Shakhar, 1990). This interpretation is supported even more convincingly by the absence of an enhanced effect of the training involving emotional images, compared to the neutral training session, once again proving that the implicit learning to ignore emotional distractors, unlike other forms of implicit learning (e.g., Gordon and Holyoak, 1983), is built on the stimuli experienced, and does not generalize to similar exemplars.

It has also been suggested that behavioral interference by emotional stimuli may be solidly associated with the affective modulation of the LPP, both of these indexing attention allocation (Weinberg and Hajcak, 2011; Weinberg et al., 2021). The present study indicates that, although LPP, Alpha-ERD, and RTs are modulated by motivational significance (emotional arousal) during the viewing of novel pictures, they are differentially affected by sustained experience with novel distractors. Additionally, in the present study, there is no significant or close-to-significant correlation between behavioral interference and the LPP/Alpha ERD (neither regarding the absolute values nor for the emotional vs. neutral differentials). Moreover, a dissociation between the LPP and behavioral interference has been described in previous habituation studies, in which behavioral emotional interference waned after only a few presentations of the same distractor, whereas the LPP amplitude was still enhanced for emotional, compared with neutral, distractors despite picture repetition (Codispoti et al., 2006, 2016; Ferrari et al., 2022). These results do not support the hypothesis that the affective modulation of the LPP indexes attentional engagement with visual stimuli uniquely associated with the subsequent behavioral interference (RT slowdown) as previously proposed by Weinberg and colleagues (Weinberg et al., 2021).

Although the LPP and the Alpha-ERD share some similarities in terms of emotional modulation (De Cesarei and Codispoti, 2011; Ferrari et al., 2022), both being unaffected by training, they may reflect partially distinct processes engaged in emotional picture processing. Indeed, no correlation was found across participants between the emotional modulation of the LPP and the Alpha-ERD in the present study, or in previous studies (De Cesarei and Codispoti, 2011; Parvaz et al., 2015; see also Li et al., 2020, for dissociation in pain perception).

From the present study, it emerges that the visual perceptual system is extremely efficient in detecting and processing novel complex natural scenes, even when they are completely irrelevant to the ongoing task and outside the attentional focus defined by the target occurrence. This initial stage of processing is very sensitive to perceptual novelty and it implies an automatic categorization process based on the motivational relevance of the stimuli, revealing what the mandatory task of the perceptual system is, namely monitoring the environment for potential threats or rewards (Donchin et al., 1978). In fact, at this stage, top-down mechanisms, such as task-relevance or experience, do not seem to play any role, as shown by the absence of any training effect on the LPP and the Alpha-ERD modulation. On the other hand, the behavioral interference depends on a more flexible mechanism.

## Data availability statement

The raw data supporting the conclusions of this article will be made available by the authors, without undue reservation.

## Ethics statement

The studies involving humans were approved by Research Ethics Board – University of Parma. The studies were conducted in accordance with the local legislation and institutional requirements. The participants provided their written informed consent to participate in this study. The individual(s) provided their written informed consent for the publication of any identifiable images or data presented in this article.

## Author contributions

VF: Conceptualization, Data curation, Formal analysis, Funding acquisition, Investigation, Methodology, Project administration, Resources, Software, Supervision, Validation, Visualization, Writing – original draft, Writing – review & editing. FC: Data curation, Investigation, Methodology, Visualization, Writing – original draft. AC: Conceptualization, Formal analysis, Methodology, Supervision, Visualization, Writing – original draft, Writing – review & editing. MC: Conceptualization, Methodology, Supervision, Visualization, Writing – original draft, Writing – review & editing.

## References

[ref1] Arana L. Melcón M. Kessel D. Hoyos S. Albert J. Carretié L.. (2022). Suppression of alpha-band power underlies exogenous attention to emotional distractors. Psychophysiology 59:e14051. doi: 10.1111/psyp.14051, PMID: 35318692 PMC9540775

[ref2] Bacigalupo Izquierdo F. Luck S. J. (2019). Lateralized suppression of alpha-band EEG activity as a mechanism of target processing. J. Neurosci. 39, 900–917. doi: 10.1523/JNEUROSCI.0183-18.2018, PMID: 30523067 PMC6382983

[ref3] Bradley M. M. (2009). Natural selective attention: orienting and emotion. Psychophysiology 46, 1–11. doi: 10.1111/j.1469-8986.2008.00702.x, PMID: 18778317 PMC3645482

[ref4] Codispoti M. De Cesarei A. Biondi S. Ferrari V. (2016). The fate of unattended stimuli and emotional habituation: behavioral interference and cortical changes. Cogn. Affect. Behav. Neurosci. 16, 1063–1073. doi: 10.3758/s13415-016-0453-0, PMID: 27557884

[ref5] Codispoti M. De Cesarei A. Ferrari V. (2023). Alpha- band oscillations and emotion: a review of studies on picture perception. Psychophysiology 60:e14438. doi: 10.1111/psyp.14438, PMID: 37724827

[ref6] Codispoti M. Ferrari V. Bradley M. M. (2006). Repetitive picture processing: autonomic and cortical correlates. Brain Res. 1068, 213–220. doi: 10.1016/j.brainres.2005.11.009 16403475

[ref7] Codispoti M. Ferrari V. Bradley M. M. (2007). Repetition and event-related potentials: distinguishing early and late processes in affective picture perception. J. Cogn. Neurosci. 19, 577–586. doi: 10.1162/jocn.2007.19.4.577 17381249

[ref8] Cohen J. (1988). Statistical power analysis for the behavioral sciences (2nd Edn). Hillsdale, NJ: Lawrence Erlbaum Associates Publishers.

[ref9] De Cesarei A. Codispoti M. (2008). Fuzzy picture processing: effects of size reduction and blurring on emotional processing. Emotion 8, 352–363. doi: 10.1037/1528-3542.8.3.352 18540751

[ref10] De Cesarei A. Codispoti M. (2011). Affective modulation of the LPP and α-ERD during picture viewing. Psychophysiology 48, 1397–1404. doi: 10.1111/j.1469-8986.2011.01204.x 21486291

[ref11] Delorme A. Makeig S. (2004). EEGLAB: an open source toolbox for analysis of single-trial EEG dynamics including independent component analysis. J. Neurosci. Methods 134, 9–21. doi: 10.1016/j.jneumeth.2003.10.009, PMID: 15102499

[ref12] Dixon M. L. Ruppel J. Pratt J. De Rosa E. (2009). Learning to ignore: acquisition of sustained attentional suppression. Psychon. Bull. Rev. 16, 418–423. doi: 10.3758/PBR.16.2.418, PMID: 19293116

[ref13] Donchin E. Ritter W. McCallum W. C. (1978). Cognitive psychophysiology: the endogenous components of the ERP. Event Related Brain Potentials Man 349:411. doi: 10.1016/B978-0-12-155150-6.50019-5

[ref14] Egeth H. E. Yantis S. (1997). Visual attention: control, representation, and time course. Annu. Rev. Psychol. 48, 269–297. doi: 10.1146/annurev.psych.48.1.269, PMID: 9046562

[ref9001] Faul F. Erdfelder E. Lang A. G. Buchner A. G. (2007). Power 3: a flexible statistical power analysis program for the social, behavioral, and biomedical sciences. Behav Res Methods. 39, 175–91. doi: 10.3758/bf03193146 17695343

[ref15] Ferrari V. Bradley M. M. Codispoti M. Lang P. J. (2011). Repetitive exposure: brain and reflex measures of emotion and attention. Psychophysiology 48, 515–522. doi: 10.1111/j.1469-8986.2010.01083.x, PMID: 20701711 PMC2982870

[ref9003] Ferrari V. Bradley M. M. Codispoti M. Lang P. J. (2015). Massed and distributed repetition of natural scenes: Brain potentials and oscillatory activity. Psychophysiology. 52, 865–872. doi: 10.1111/psyp.12424, PMID: 25847093

[ref16] Ferrari V. Bruno N. Chattat R. Codispoti M. (2017). Evaluative ratings and attention across the life span: emotional arousal and gender. Cognit. Emot. 31, 552–563. doi: 10.1080/02699931.2016.1140020, PMID: 26864052

[ref17] Ferrari V. Canturi F. Codispoti M. (2022). Stimulus novelty and emotionality interact in the processing of visual distractors. Biol. Psychol. 167:108238. doi: 10.1016/j.biopsycho.2021.108238, PMID: 34864068

[ref18] Ferrari V. Codispoti M. Cardinale R. Bradley M. M. (2008). Directed and motivated attention during processing of natural scenes. J. Cogn. Neurosci. 20, 1753–1761. doi: 10.1162/jocn.2008.20121 18370595

[ref19] Ferrari V. Mastria S. Codispoti M. (2020). The interplay between attention and long-term memory in affective habituation. Psychophysiology 57:e13572. doi: 10.1111/psyp.13572, PMID: 32239721

[ref20] Folk C. L. (2015). “Controlling spatial attention: lessons from the lab and implications for everyday life” in The handbook of attention. eds. Fawcett J. M. Risko E. F. Kingstone A. (Cambridge, MA: MIT Press), 3–25.

[ref21] Gati I. Ben-Shakhar G. (1990). Novelty and significance in orientation and habituation: a feature-matching approach. J. Exp. Psychol. Gen. 119, 251–263. doi: 10.1037/0096-3445.119.3.251, PMID: 2145391

[ref22] Gordon P. C. Holyoak K. J. (1983). Implicit learning and generalization of the" mere exposure" effect. J. Pers. Soc. Psychol. 45:492. doi: 10.1037/0022-3514.45.3.492, PMID: 33204439

[ref23] Gottlieb J. (2012). Attention, learning, and the value of information. Neuron 76, 281–295. doi: 10.1016/j.neuron.2012.09.034, PMID: 23083732 PMC3479649

[ref24] Gratton G. Coles M. G. Donchin E. (1983). A new method for off-line removal of ocular artifact. Electroencephalogr. Clin. Neurophysiol. 55, 468–484. doi: 10.1016/0013-4694(83)90135-9 6187540

[ref25] Hamm A. O. Schupp H. T. Weike A. I. (2003). Motivational Organization of Emotions: Autonomic changes, cortical responses, and reflex modulation. Oxford: Oxford University Press.

[ref26] Hartikainen K. M. Ogawa K. H. Knight R. T. (2000). Transient interference of right hemispheric function due to automatic emotional processing. Neuropsychologia 38, 1576–1580. doi: 10.1016/S0028-3932(00)00072-5, PMID: 11074080

[ref27] Herrmann C. S. Grigutsch M. Busch N. A. (2005). “11 EEG oscillations and wavelet analysis” in Event-related potentials: A methods handbook. ed. Handy T. C. (Cambridge, MA: MIT Press), 229.

[ref28] Junghofer M. Elbert T. Tucker D. M. Rockstroh B. (2000). Statistical control of artifacts in dense array EEG/MEG studies. Psychophysiology 37, 523–532. doi: 10.1111/1469-8986.3740523, PMID: 10934911

[ref29] Kelley T. A. Yantis S. (2009). Learning to attend: effects of practice on information selection. J. Vis. 9, 1–18. doi: 10.1167/9.7.16 19761331 PMC3124869

[ref30] Kelley T. A. Yantis S. (2010). Neural correlates of learning to attend. Front. Hum. Neurosci. 4:216. doi: 10.3389/fnhum.2010.00216, PMID: 21120137 PMC2991198

[ref31] Lang P. J. Bradley M. M. Cuthbert B. N. (2008). International affective picture system (IAPS): Affective ratings of pictures and instruction manual. Technical Report A-8.. Gainesville: University of Florida.

[ref32] Li Q. Joo S. J. Yeatman J. D. Reinecke K. (2020). Controlling for participants’ viewing distance in large-scale, psychophysical online experiments using a virtual chinrest. Sci. Rep. 10:904. doi: 10.1038/s41598-019-57204-1 31969579 PMC6976612

[ref33] MacNamara A. Hajcak G. (2009). Anxiety and spatial attention moderate the electrocortical response to aversive pictures. Neuropsychologia 47, 2975–2980. doi: 10.1016/j.neuropsychologia.2009.06.026, PMID: 19576234

[ref34] Mastria S. Ferrari V. Codispoti M. (2017). Emotional picture perception: repetition effects in free-viewing and during an explicit categorization task. Front. Psychol. 8:1001. doi: 10.3389/fpsyg.2017.01001 28725202 PMC5495866

[ref35] Micucci A. Ferrari V. De Cesarei A. Codispoti M. (2020). Contextual modulation of emotional distraction: attentional capture and motivational significance. J. Cogn. Neurosci. 32, 621–633. doi: 10.1162/jocn_a_01505, PMID: 31765599

[ref36] Most S. B. Smith S. D. Cooter A. B. Levy B. N. Zald D. H. (2007). The naked truth: positive, arousing distractors impair rapid target perception. Cognit. Emot. 21, 964–981. doi: 10.1080/02699930600959340

[ref37] Mulckhuyse M. (2018). The influence of emotional stimuli on the oculomotor system: a review of the literature. Cogn. Affect. Behav. Neurosci. 18, 411–425. doi: 10.3758/s13415-018-0590-8, PMID: 29633198

[ref38] Murphy J. Devue C. Corballis P. M. Grimshaw G. M. (2020). Proactive control of emotional distraction: evidence from EEG alpha suppression. Front. Hum. Neurosci. 14:318. doi: 10.3389/fnhum.2020.00318 33013338 PMC7461792

[ref9002] O’Brien B. C. Harris I. B. Beckman T. J. Reed D. A. Cook D. A. (2014). Standards for reporting qualitative research: a synthesis of recommendations. Acad Med. 89, 1245–51. doi: 10.1097/ACM.0000000000000388, PMID: 24979285

[ref39] Öhman A. (1992). “Orientingand attention: preferred preattentive processing of potentially phobic stimuli” in Attention and information processing in infants and adults. eds. Campbell B. A. Hayne H. Richardson R. (Hillsdale: Erlbaum), 263–295.

[ref40] Parvaz M. A. Moeller S. J. Goldstein R. Z. Proudfit G. H. (2015). Electrocortical evidence of increased post-reappraisal neural reactivity and its link to depressive symptoms. Soc. Cogn. Affect. Neurosci. 10, 78–84. doi: 10.1093/scan/nsu027, PMID: 24526188 PMC4994842

[ref41] Peyk P. De Cesarei A. Junghöfer M. (2011). ElectroMagnetoEncephaloGraphy software (EMEGS): overview and integration with other EEG/MEG toolboxes. Comput. Intell. Neurosci. 2011:861705. doi: 10.1155/2011/861705, PMID: 21577273 PMC3090751

[ref42] Roach B. J. Mathalon D. H. (2008). Event-related EEG time-frequency analysis: an overview of measures and an analysis of early gamma band phase locking in schizophrenia. Schizophr. Bull. 34, 907–926. doi: 10.1093/schbul/sbn093 18684772 PMC2632478

[ref43] Schimmack U. (2005). Attentional interference effects of emotional pictures: threat, negativity, or arousal? Emotion 5, 55–66. doi: 10.1037/1528-3542.5.1.55, PMID: 15755219

[ref44] Schneider W. Eschman A. Zuccolotto A. (2002). E-prime reference guide. Pittsburg, PA: Psychology Software Tools

[ref9004] Schubring D. Schupp H. T. (2019). Affective picture processing: Alpha- and lower beta-band desynchronization reflects emotional arousal. Psychophysiology, e13386. doi: 10.1111/psyp.13386, PMID: 31026079

[ref45] Schubring D. Schupp H. T. (2021). Emotion and brain oscillations: high arousal is associated with decreases in alpha- and lower beta-band power. Cereb. Cortex 31, 1597–1608. doi: 10.1093/cercor/bhaa312, PMID: 33136146

[ref46] Schupp H. T. Flaisch T. Stockburger J. Junghöfer M. (2006). Emotion and attention: event-related brain potential studies. Prog. Brain Res. 156, 31–51. doi: 10.1016/S0079-6123(06)56002-9 17015073

[ref47] Tallon-Baudry C. Bertrand O. Delpuech C. Pernier J. (1997). Oscillatory gamma-band (30–70 Hz) activity induced by a visual search task in humans. J. Neurosci. 17, 722–734. doi: 10.1523/JNEUROSCI.17-02-00722.1997, PMID: 8987794 PMC6573221

[ref48] Vatterott D. B. Mozer M. C. Vecera S. P. (2018). Rejecting salient distractors: generalization from experience. Atten. Percept. Psychophys. 80, 485–499. doi: 10.3758/s13414-017-1465-8, PMID: 29230673

[ref49] Vatterott D. B. Vecera S. P. (2012). Experience-dependent attentional tuning of distractor rejection. Psychon. Bull. Rev. 19, 871–878. doi: 10.3758/s13423-012-0280-4, PMID: 22696250

[ref50] Vecera S. P. Cosman J. D. Vatterott D. B. Roper Z. J. (2014). “The control of visual attention: toward a unified account” in Psychology of learning and motivation, vol. 60 (Cambridge, MA: Academic Press), 303–347.

[ref51] Weinberg A. Correa K. A. Stevens E. S. Shankman S. A. (2021). The emotion-elicited late positive potential is stable across five testing sessions. Psychophysiology 58:e13904. doi: 10.1111/psyp.13904, PMID: 34292629

[ref52] Weinberg A. Hajcak G. (2011). The late positive potential predicts subsequent interference with target processing. J. Cogn. Neurosci. 23, 2994–3007. doi: 10.1162/jocn.2011.21630 21268668

[ref53] Wendt J. Weike A. I. Lotze M. Hamm A. O. (2011). The functional connectivity between amygdala and extrastriate visual cortex activity during emotional picture processing depends on stimulus novelty. Biol. Psychol. 86, 203–209. doi: 10.1016/j.biopsycho.2010.11.009, PMID: 21130141

